# Mitochondrial calcium uniporter is essential for hearing and hair cell preservation in congenic FVB/NJ mice

**DOI:** 10.1038/s41598-021-88841-0

**Published:** 2021-05-06

**Authors:** Mayakannan Manikandan, Steven Walker, Aditi R. Deshmukh, Elizabeth Perea, Danqi Wang, Kumar N. Alagramam, Ruben Stepanyan

**Affiliations:** 1 Department of Otolaryngology–Head and Neck Surgery, School of Medicine, Case Western Reserve University and University Hospitals Cleveland Medical Center, 11100 Euclid Ave., Cleveland, OH 44106 USA; 2 Swagelok Center for Surface Analysis of Materials, Case Western Reserve University, Cleveland, OH 44106 USA; 3 Department of Neurosciences, School of Medicine, Case Western Reserve University, Cleveland, OH 44106 USA; 4 Department of Genetics and Genome Sciences, School of Medicine, Case Western Reserve University, Cleveland, OH 44106 USA

**Keywords:** Mitochondria, Development, Experimental models of disease, Mouse

## Abstract

Mitochondrial Ca^2+^ regulates a wide range of cell processes, including morphogenesis, metabolism, excitotoxicity, and survival. In cochlear hair cells, the activation of mechano-electrical transduction and voltage-gated Ca^2+^ channels result in a large influx of Ca^2+^. The intracellular rise in Ca^2+^ is partly balanced by the mitochondria which rapidly uptakes Ca^2+^ via a highly selective channel comprised of the main pore-forming subunit, the mitochondrial Ca^2+^ uniporter (MCU), and associated regulatory proteins. MCU thus contributes to Ca^2+^ buffering, ensuring cytosolic homeostasis, and is posited to have a critical role in hair cell function and hearing. To test this hypothesis, Ca^2+^ homeostasis in hair cells and cochlear function were investigated in FVB/NJ mice carrying the knockout allele of *Mcu* (*Mcu*^+*/−*^ or *Mcu*^*−/−*^). The *Mcu* knockout allele, which originated in C57BL/6 strain cosegregated along with *Cdh23*^*ahl*^ allele to the FVB/NJ strain, due to the close proximity of these genes. Neither *Mcu*^+*/−*^ nor *Mcu*^*−/−*^ genotypes affected cochlear development, morphology, or Ca^2+^ homeostasis of auditory hair cells in the first two postnatal weeks. However, *Mcu*^*−*/*−*^ mice displayed high-frequency hearing impairment as early as 3 weeks postnatal, which then progressed to profound hearing loss at all frequencies in about 6 months. In *Mcu*^+*/−*^ mice, significantly elevated ABR thresholds were observed at 6 months and 9 months of age only at 32 kHz frequency. In three-month-old *Mcu*^*−/−*^ mice, up to 18% of the outer hair cells and occasionally some inner hair cells were missing in the mid-cochlear region. In conclusion, mitochondrial Ca^2+^ uniporter is not required for the development of cochlea in mice, but is essential for hearing and hair cell preservation in congenic FVB/NJ mice.

## Introduction

Acquired hearing impairment is mostly associated with deficiency or loss of cochlear cells laden with mitochondria: the inner and outer hair cells (HCs), the spiral ganglion neurons (SGNs), and the cells of the stria vascularis^1,2^. Predictably, evidence implicates that mitochondrial dysfunction is a lead cause in several nonsyndromic, syndromic, and acquired forms of deafness^3,4^. Moreover, mitochondrial DNA mutations (mtDNA) contribute to more than 5% of post-lingual non-syndromic hearing impairment in the Caucasian and Asian populations^5,6^. Mutations in the mtDNA or nuclear DNA, which affect mitochondrial function, could result in increased oxidative damage and associated loss of HCs, SGNs, or cells of stria vascularis^7,8^. Like in many other cell types in an organism, mitochondria in the cochlear cells are responsible for vital cellular functions, including energy production, apoptosis, cell signaling, and calcium storage. These functions are dependent on the ability of mitochondria to modulate Ca^2+^ levels^9–15^. Particularly, Ca^2+^ uptake via mitochondrial calcium uniporter (MCU) is critical for the rapid buffering of increases in intracellular Ca^2+^ loads; an increase in mitochondrial calcium regulates ATP production to fuel Ca^2+^ extrusion from the cell^16^. Surprisingly, while slightly smaller in size, mice lacking MCU have a normal life-span and exhibit no adverse phenotype when unchallenged^17^. These mice were generated on a mixed C57BL/6-CD1 (B6-CD1) background^17^ and the loss of MCU was not conclusively shown to affect hearing^18^. Importantly, due to their genetic predisposition, both the C57BL/6 and CD1 strains show progressive hearing loss^19,20^, making it impossible to discern the pathogenic contribution of *Mcu* loss above incumbent hearing impairment. Therefore, a "good hearing" strain, such as the CBA/CaJ or FVB/NJ^19,21,22^ is necessary to transcend the potentially confounding issues associated with the B6-CD1 background. For this purpose, herein, we used marker-assisted breeding, or speed congenics, to transfer the *Mcu* knockout allele to the FVB/NJ background. Since the *Mcu* knockout allele is very closely located to the *Cdh23*^*ahl*^ allele, which is linked to age-related hearing loss, they cosegregated to the FVB/NJ background. Consequently, the FVB/NJ mice homozygous for the knockout allele of *Mcu* (*Mcu*^*−/−*^) were also homozygous for the C57BL/6 derived *Cdh23*^*ahl*^ allele. We found that the *Mcu*^*−/−*^ mice develop substantial hearing loss at one month across all frequencies tested, experience degeneration of cochlear hair cells at 3 months, and are profoundly deaf by 6 months of age. This study demonstrates that *Mcu* is essential for hearing preservation in congenic FVB/NJ mice.

## Results

### Strain-specific effects of *Mcu*^*−/−*^ and *Mcu*^+*/−*^ on hearing in mice

**Figure 1:**
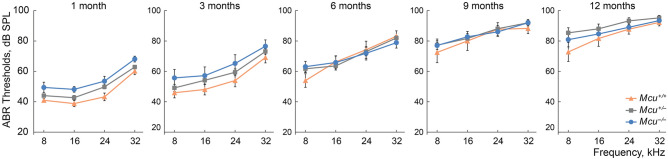
Mice on the mixed B6-CD1 background are predisposed to rapidly progressing hearing loss at all frequencies. Auditory brainstem responses (ABRs) were recorded from the *Mcu*^+*/*+^, *Mcu*^+*/−*^, and *Mcu*^*−/−*^ mice on the mixed B6-CD1 background at various time points. *Mcu*^*−/−*^ mice show a trend toward slightly elevated ABR thresholds up to three months of age. Although analyses did not show any statistically significant differences among *Mcu*^+*/*+^, *Mcu*^+*/−*^ and *Mcu*^*−/−*^ mice, as assessed using two way ANOVA followed by Bonferroni multiple comparison test. Not every mouse was tested at each time point. Number of mice (*Mcu*^+*/*+^, *Mcu*^+*/−*^, *Mcu*^*−/−*^): 1 month (23, 32, 11); 3 months (17, 30, 12); 6 months (12, 28, 19); 9 months (10, 25, 19); 12 months (9, 33, 14).

**Figure 2:**
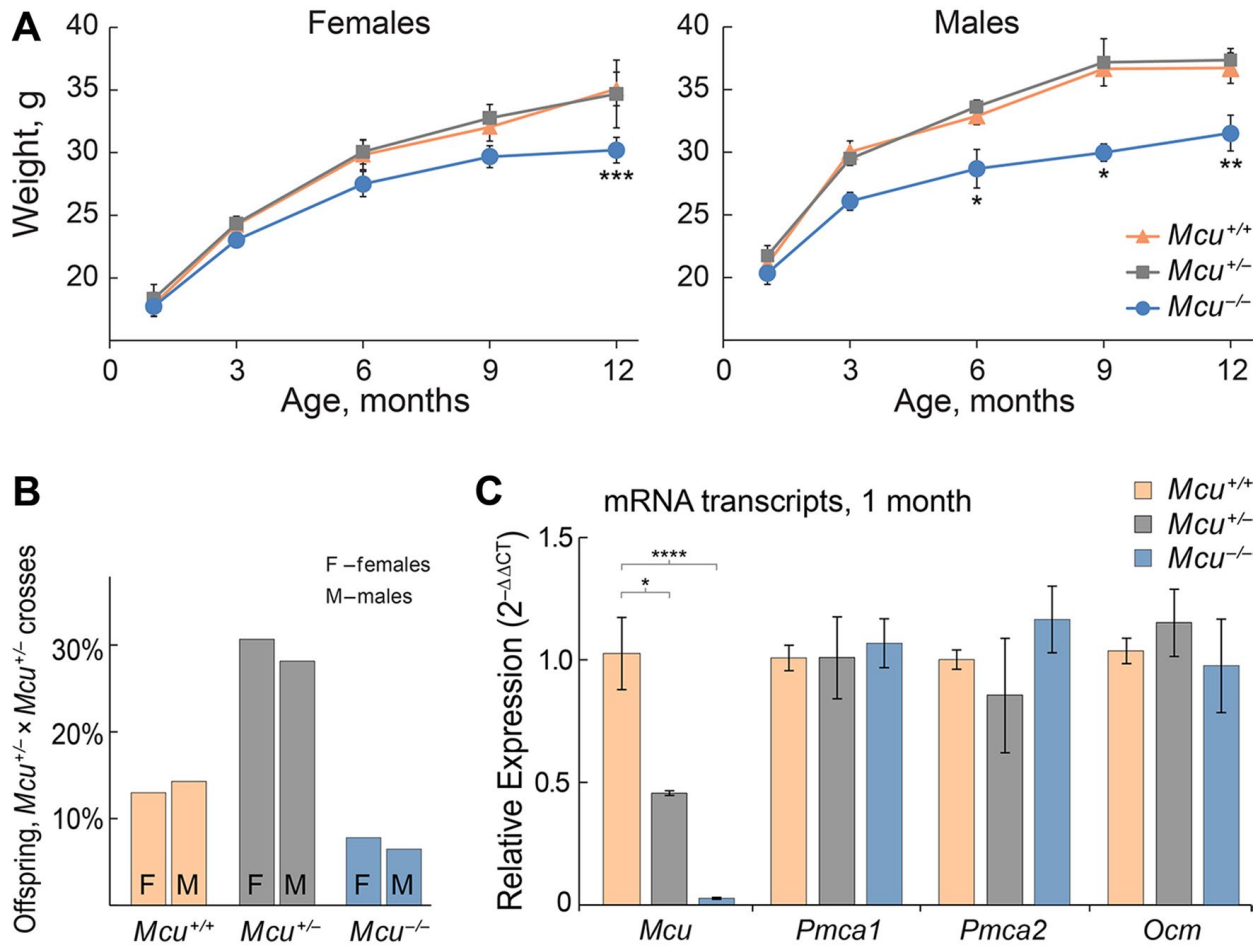
*Mcu*^*−/−*^ mice on the FVB/NJ background are smaller in size. (**A**) *Mcu*^*−/−*^ mice have smaller body sizes and reduced body weight in comparison to *Mcu*^+*/−*^ and *Mcu*^+*/*+^ littermates. Number of female mice (*Mcu*^+*/*+^, *Mcu*^+*/−*^, *Mcu*^*−/−*^): 1 month (12, 11, 11); 3 months (12, 21, 11); 6 months (7, 15, 9); 9 months (10, 17, 13); 12 months (5, 7, 5). Number of male mice (*Mcu*^+*/*+^, *Mcu*^+*/−*^, *Mcu*^*−/−*^): 1 month (12, 15, 6); 3 months (15, 17, 6); 6 months (8, 22, 9); 9 months (5, 12, 7); 12 months (3, 16, 3). (**B**) Percentage of female (n = 39) and male (n = 37) offspring obtained from *Mcu*^+*/−*^ × *Mcu*^+*/−*^ crosses (8 litters). (**C**) Relative quantitation of the mRNA levels of *Mcu*, *Pmca1*, *Pmca2*, and *Ocm* in the cochlea of FVB/NJ mice of *Mcu*^*−/−*^ and *Mcu*^+*/−*^ genotypes as compared to those of *Mcu*^+*/*+^ littermates. Cochleae obtained from 6 animals each of *Mcu*^+*/*+^, *Mcu*^+*/−*^, *Mcu*^*−/−*^ genotypes at 1 month of age were used for studying gene expression. *p < 0.05, **p < 0.01, ***p < 0.001, **** *p* < 0.0001, Two way ANOVA followed by Bonferroni multiple comparison tests.

**Figure 3:**
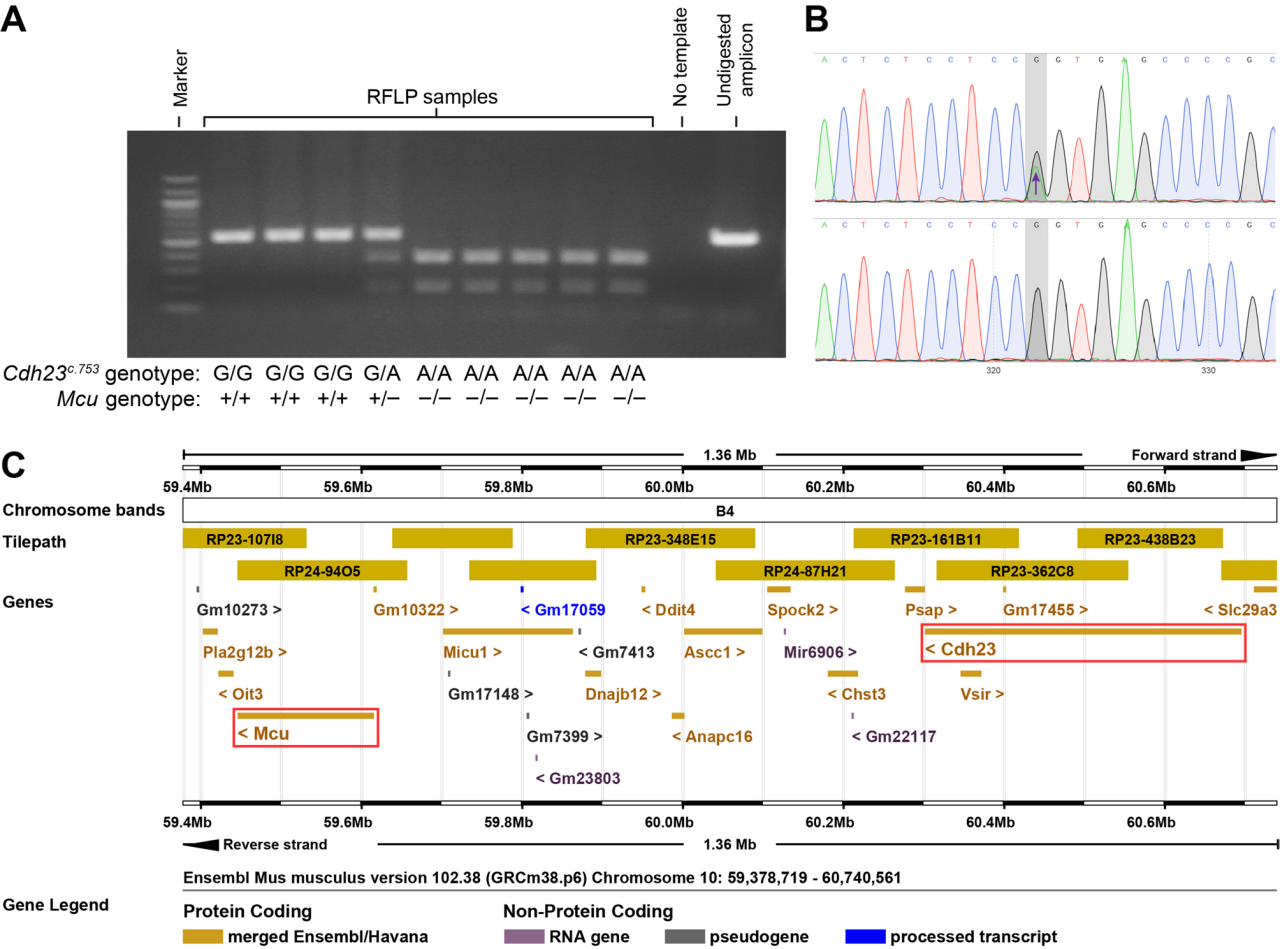
PCR and restriction fragment length polymorphism (RFLP) based genotyping of *Cdh23*^*c.753A/G*^ in FVB/NJ *Mcu*^*−/−*^ mice. (**A**) A representative electropherogram showing the PCR amplicons resolved in a 1.5% agarose gel following digestion with *BsrI*. The *Cdh23*^*c.753G*^ allele renders the binding site for *BsrI* inaccessible to digest the PCR amplicon of 534 bp, while the *Cdh23c.*^*753A*^ allele creates a *BsrI* binding site resulting in fragments of 350 bp and 184 bp. (**B**) DNA sequence chromatogram validates the PCR–RFLP method to genotype *Cdh23*^*c.753A/G*^ polymorphism. The top panel shows a heterozygous *Cdh23*^*c.753A/G*^ allele previously identified by restriction with *BsrI*. The bottom panel shows a homozygous for *Cdh23*^*c.753G*^ sample unaffected by *BsrI*. (**C**) Ensembl-based analysis shows genes in a 1.36 Mb region of mouse Chr.10qB4. The genes *Mcu* and *Cdh23* (red boxes) are closely located with an intergenic distance of ~ 686 kb.

To determine a potential role of MCU in mammalian hearing, we first assessed the auditory brainstem responses (ABRs) of wild type (*Mcu*^+*/*+^), heterozygous (*Mcu*^+*/−*^), and homozygous (*Mcu*^*−/−*^) mice on the mixed B6-CD1 background^17^ at various ages (Fig. 1). *Mcu*^*−/−*^ mice showed a modest elevation in ABR thresholds at one month of age when compared to *Mcu*^+*/−*^ or *Mcu*^+*/*+^ littermates. However, this early difference in threshold elevation in the *Mcu*^*−/−*^ mice was followed by concomitant elevation in ABR thresholds in the *Mcu*^+*/−*^ and *Mcu*^+*/*+^ littermates, starting from 3 months onward (Fig. 1). Our data confirm the obscuring genetic effect of the B6-CD1 background and to rectify this problem, we used marker-assisted breeding to transfer the *Mcu* knockout allele to the FVB/NJ and CBA/CaJ backgrounds. CBA/CaJ *Mcu*^+*/−*^ × CBA/CaJ *Mcu*^+*/−*^ crosses did not yield any CBA/CaJ *Mcu*^*−/−*^ offspring and henceforth all experiments were conducted using the FVB/NJ strain. Other than being smaller (Fig. 2A), young and old FVB/NJ *Mcu*^*−/−*^ mice were indistinguishable from their *Mcu*^+*/−*^ or *Mcu*^+*/*+^ littermates. FVB/NJ *Mcu*^+*/−*^ × FVB/NJ *Mcu*^+*/−*^ crosses produced only 14.5% of an approximately equal number of *Mcu*^*−/−*^ males and females, while *Mcu*^+*/−*^ mice comprised 57.9% (Fig. 2B). The small body size and reduced number of *Mcu*^*−/−*^ offspring in the FVB/NJ background is on par with the observation reported in the mixed B6-CD1 background^17^. For confirmation, we first quantified the transcript levels of *Mcu* in the cochlea of FVB/NJ mice of all three genotypes. Indeed, the mRNA levels of *Mcu* were reduced significantly, by more than 50% in the *Mcu*^+*/−*^ mice and by 98% in *Mcu*^*−/−*^ mice compared to the *Mcu*^+*/*+^ littermates (Fig. 2C). Additionally, the relative transcript levels of the predominant PMCA pumps present in cochlear hair cells (*Pmca1* and *Pmca2*) and the calcium-binding protein oncomodulin (*Ocm*) were similar across all three genotypes (Fig. 2C) indicating that *Mcu* expression-deficiency is not compensated by the overexpression of the tested Ca^2+^ regulators. As the *Mcu* knockout allele was generated using blastocysts of C57BL/6 mice^17^, we probed the FVB/NJ mice backcrossed for the *Mcu* knockout allele for a well-known polymorphism that causes age-related hearing loss (AHL) in C57BL/6 mice—the *Cdh23*^*ahl*^ allelic variant^23^. Among a panel of 51 mice tested (Fig. 3A,B), all *Mcu*^*−/−*^ mice (n = 22) were also found to be *Cdh23*^*ahl/ahl*^ (homozygous for *Cdh23*^*c.753A*^), the *Mcu*^+*/*+^ mice (n = 12) were *Cdh23*^+*/*+^ (homozygous for *Cdh23*^*c.*753G^), and *Mcu*^+*/−*^ mice (n = 17) were *Cdh23*^+*/ahl*^ (*Cdh23*^*c.753G/A*^). Genomic coordinate analyses of *Mcu* and *Cdh23* in Ensembl genome browser identified the close proximity of these genes in Chr.10qB4, with an intergenic distance of ~ 686 kb (Fig. 3C). Thus, the *Cdh23*^*ahl*^ allele is essentially inseparable from the *Mcu* knockout allele by conventional breeding, and the FVB/NJ *Mcu*^*−/−*^ mice are homozygous for *Cdh23*^*ahl*^.

**Figure 4:**
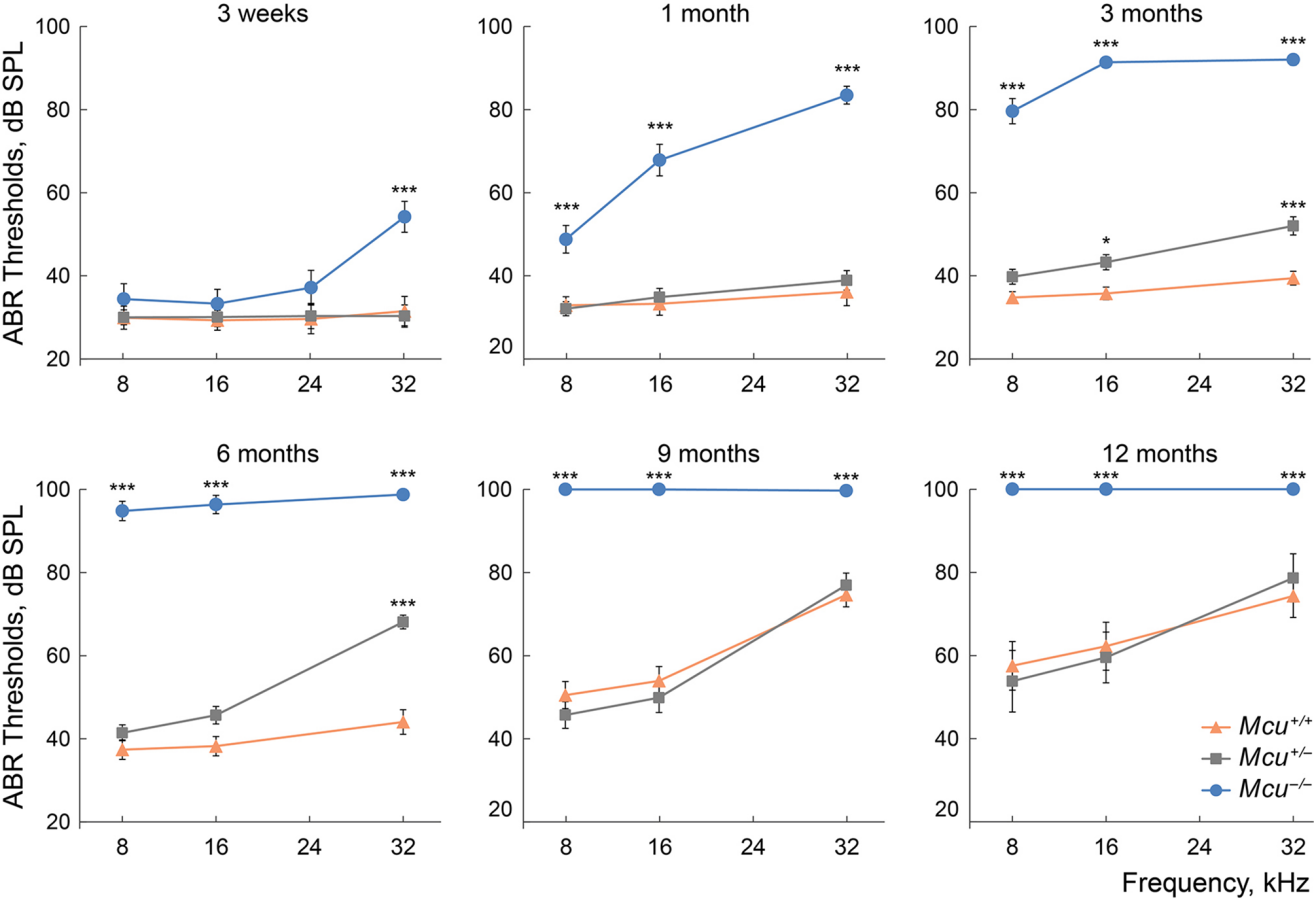
Hearing loss in *Mcu*^*−/−*^ mice on FVB/NJ background. Early-onset high-frequency hearing loss rapidly progresses to profound deafness with age in FVB/NJ *Mcu*^*−/−*^ mice. Auditory brainstem responses were recorded from the *Mcu*^*−/−*^, *Mcu*^+*/−*^, and *Mcu*^+*/*+^ mice of FVB/NJ background, at various time points. Also, note the modest high-frequency hearing loss starting from 3 months of age in *Mcu*^+*/−*^ mice. *p < 0.05, ***p < 0.001, two-way ANOVA followed by Bonferroni multiple comparison test. Not every mouse was tested at each time point. Same mice were used as in Fig. 2A plus 8 *Mcu*^+*/*+^, 21 *Mcu*^+*/−*^, and 11 *Mcu*^*−/−*^ 3-week-old mice.

Following hearing onset, the congenic FVB/NJ *Mcu*^*−/−*^ mice displayed normal ABR thresholds at low- to mid-frequencies and a 10–15 dB SPL threshold elevation at a high (32 kHz) frequency compared to the *Mcu*^+*/−*^ or *Mcu*^+*/*+^ littermates (Fig. 4). At 1 month, it became apparent that the FVB/NJ *Mcu*^*−/−*^ mice could not preserve the hearing sensitivity they displayed around onset; threshold elevation was significant at 8, 16, and 32 kHz. This trend continued at 3 months, leading to profound hearing loss (Fig. 4). Also, ABR threshold elevations at 16 and 32 kHz in 3 month old heterozygous *Mcu*^+*/−*^ mice, followed by a further decrease in hearing sensitivity at 32 kHz at 6 months, were observed relative to the corresponding thresholds in the *Mcu*^+*/*+^ littermates (Fig. 4). This observation suggests haploinsufficiency, wherein one copy of *Mcu* results in reduced mRNA levels (Fig. 2C) and is not sufficient to preserve hearing.

### Development of the cochlea in *Mcu*^−/−^ mice

**Figure 5:**
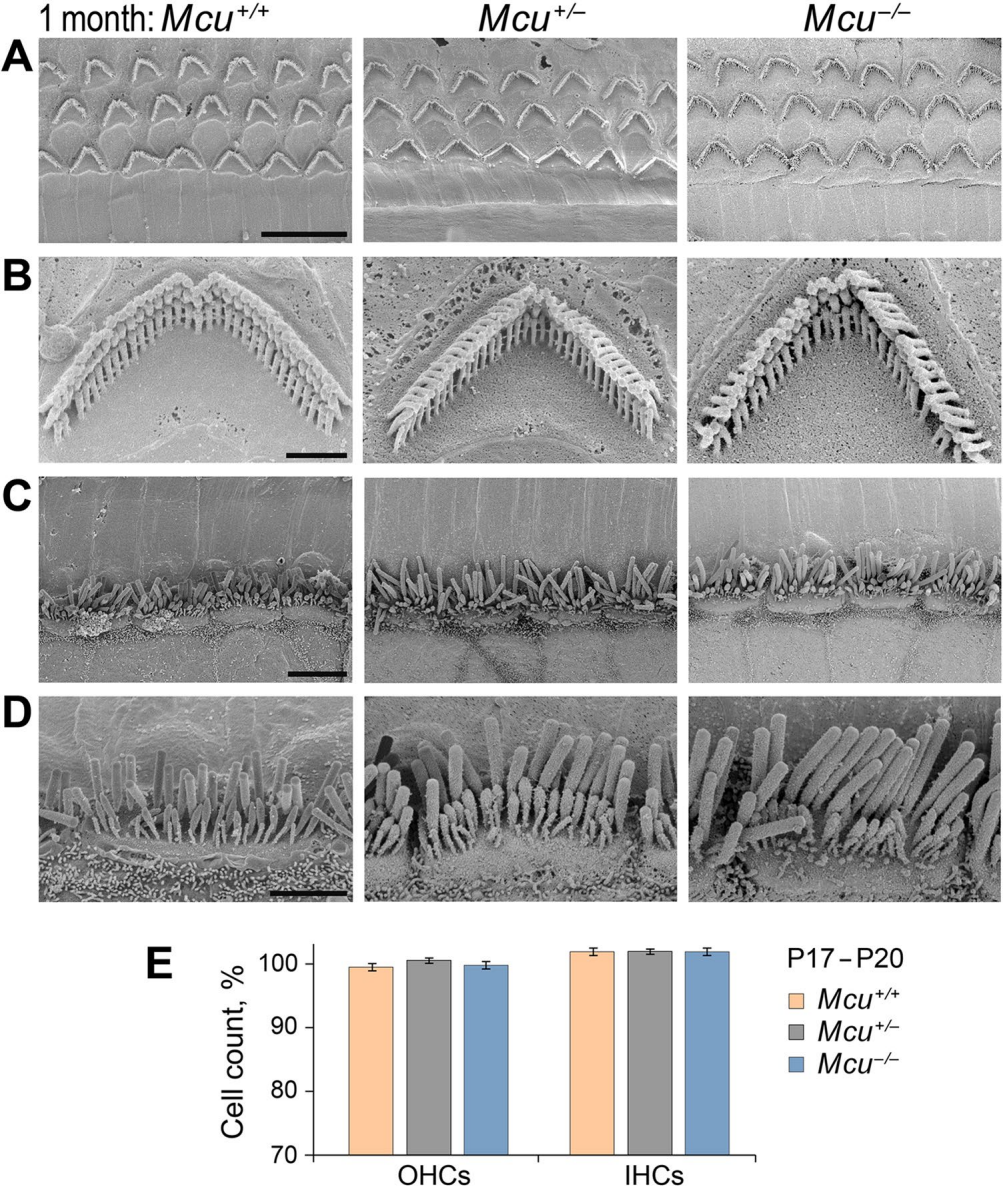
Cochlear hair cells in young FVB/NJ *Mcu*^+*/*+^, *Mcu*^+*/−*^, and *Mcu*^*−/−*^ mice. (**A**–**D**) Representative scanning electron micrographs of cochlear hair cells from 1-month-old FVB/NJ *Mcu*^+*/*+^, *Mcu*^+*/−*^, and *Mcu*^*−/−*^ mice. (**A**) The typical three-row arrangement of OHCs, (**B**) single OHC stereocilia at high magnification, (**C**) the single row of IHCs, and (**D**) single IHC stereocilia at high magnification. (**E**) The number of hair cells in the mid-basal cochlear turn at P17–20. Kruskal–Wallis analysis with Dunn’s multiple comparison test did not show any statistically significant differences between groups. The number of mice is 9 (*Mcu*^+*/*+^), 21 (*Mcu*^+*/−*^), and 9 (*Mcu*^*−/−*^). Scale bars are (in µm): 10 (**A**); 1 (**B**); 5 (**C**); 2 (**D**).

As mentioned above, FVB/NJ *Mcu*^*−/−*^ mice were smaller in size, but otherwise normal. The relatively normal ABR thresholds in the *Mcu*^*−/−*^ mice at 3 weeks of age (Fig. 4) indicated that the development of the cochlea and the sequential maturation of the peripheral auditory sense organ is not dependent on the MCU. We examined the cochlear hair cells (HCs) in *Mcu*^*−/−*^ mice and their littermate controls. The number of HCs at the time following the onset of the hearing was similar in *Mcu*^*−/−*^ mice compared to the *Mcu*^+*/−*^ and *Mcu*^+*/*+^ littermates (Fig. 5E). Electron micrographs of HCs or their stereocilia from the *Mcu*^*−/−*^ mice at one month (Fig. 5A–D) showed no structural defects.

**Figure 6:**
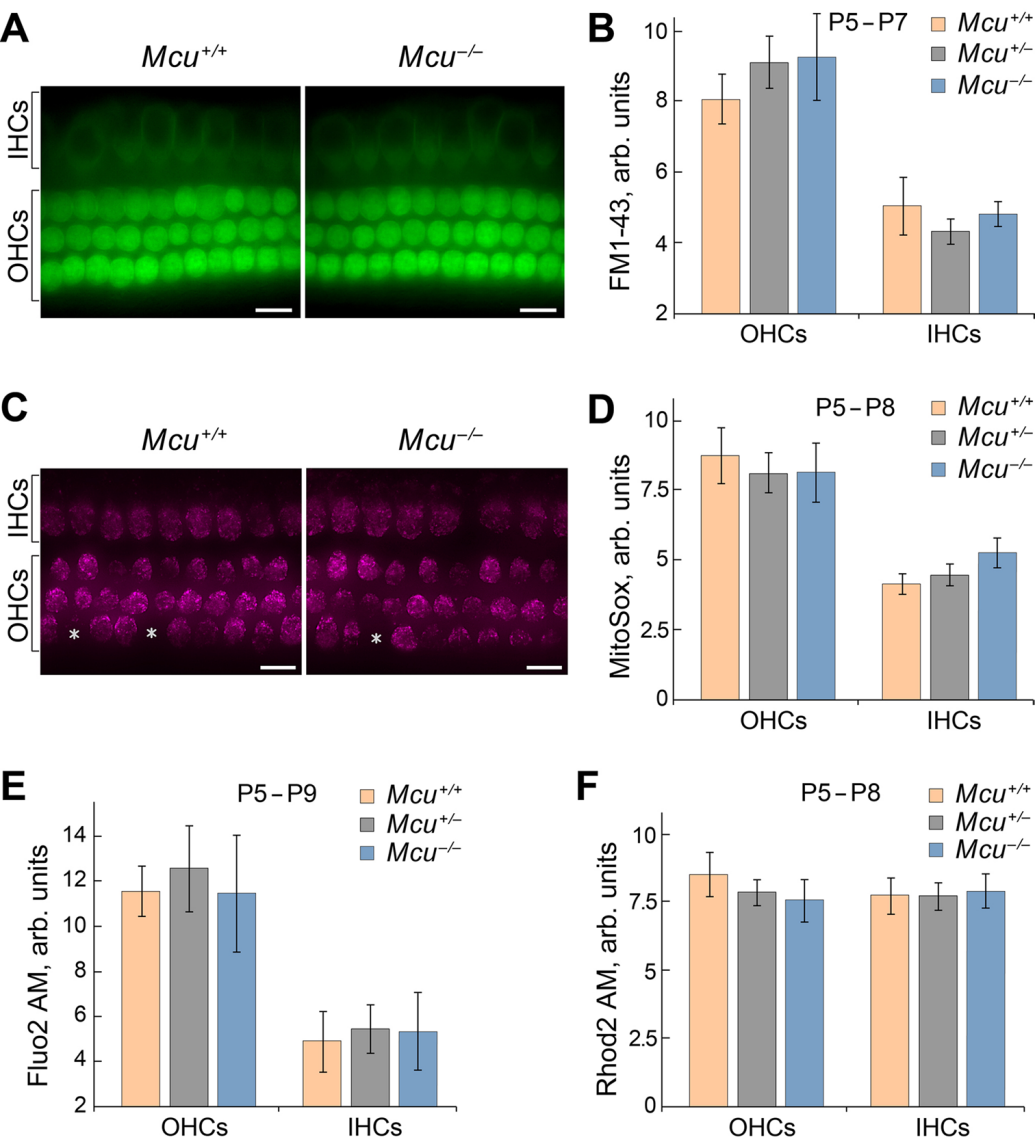
*Mcu* is not required for the development of hair cells. Results obtained in vitro demonstrate that mechanotransduction, Ca^2+^ levels, and mitochondria superoxide activity are not altered in the cochlear hair cells of *Mcu*^*−/−*^ mice. (**A**) Representative images show FM1-43 uptake in hair cells of *Mcu*^+*/*+^, *Mcu*^+*/−*^* or Mcu*^*−/−*^ mice (mid-basal turn). (**B**) The fluorescence intensity of FM1-43 in hair cells is not significantly different between the groups, indicating the normal functioning of MET channels in *Mcu*^*−/−*^ mice. (**C**) Representative images showing the MitoSOX indicator levels in hair cells of *Mcu*^+*/*+^or *Mcu*^*−/−*^. Asterisks show *Mcu*^+*/*+^ and *Mcu*^*−/−*^ OHCs, which were excluded from statistical analysis due to no detectable superoxide activity. (**D**) The fluorescence intensity of MitoSOX in hair cells of *Mcu*^*−/−*^ mice is similar to that of the *Mcu*^+*/*+^ mice indicating that there is no difference in oxidative activity caused by cellular superoxide buildup. (**E**,**F**) The fluorescence intensity of intracellular (Fluo-2 AM) and mitochondrial (Rhod-2 AM) Ca^2+^ indicators in hair cells of *Mcu*^+*/*+^, *Mcu*^+*/−*^ and *Mcu*^*−/−*^ mice were not significantly different. Data represented herein are from mice of age P5–P9. Two-way ANOVA analysis did not show any statistically significant differences between *Mcu*^+*/*+^, *Mcu*^+*/−*^* or Mcu*^*−/−*^ groups. The number of mice for all groups was greater than 5. Scale bars in (**A**) and (**C**) are 12 µm.

Mitochondrial Ca^2+^ uptake is thought to be essential for the functioning of HCs. We examined whether (i) the calcium levels are affected in HCs of FVB/NJ *Mcu*^*−/−*^ mice at postnatal (P) day P5–P8, after they acquire mechanosensitivity^24–26^, and whether (ii) the Ca^2+^ flow via mechano-electrical transduction (MET) channels will be buffered by HC mitochondria differently in *Mcu*^*−/−*^ mice compared to the controls. Initially, the MET function was estimated using FM1-43, a fluorescent molecule that serves as an indicator of mechanotransduction channel function. FM1-43 uptake by HCs of *Mcu*^*−/−*^ mice demonstrated no significant difference in MET activity as compared to the *Mcu*^+*/−*^ and *Mcu*^+*/*+^ littermates (Fig. 6A,B). Likewise, the Ca^2+^ levels in HCs of *Mcu*^*−/−*^, *Mcu*^+*/−*^ and *Mcu*^+*/*+^ mice were similar (Fig. 6E,F). Mitochondrial Ca^2+^ uptake may regulate reactive oxygen species (ROS) production^27,28^. Within the mitochondria, the primary source of ROS is superoxide^29–31^. Therefore, we analyzed the superoxide levels in the mitochondria of HCs in *Mcu*^*−/−*^ mice, and found them to be similar to those in *Mcu*^+*/−*^ and *Mcu*^+*/*+^ mice (Fig. 6C,D).

### Loss of cochlear hair cell stereocilia in three month old Mcu^***−/−***^ mice

**Figure 7:**
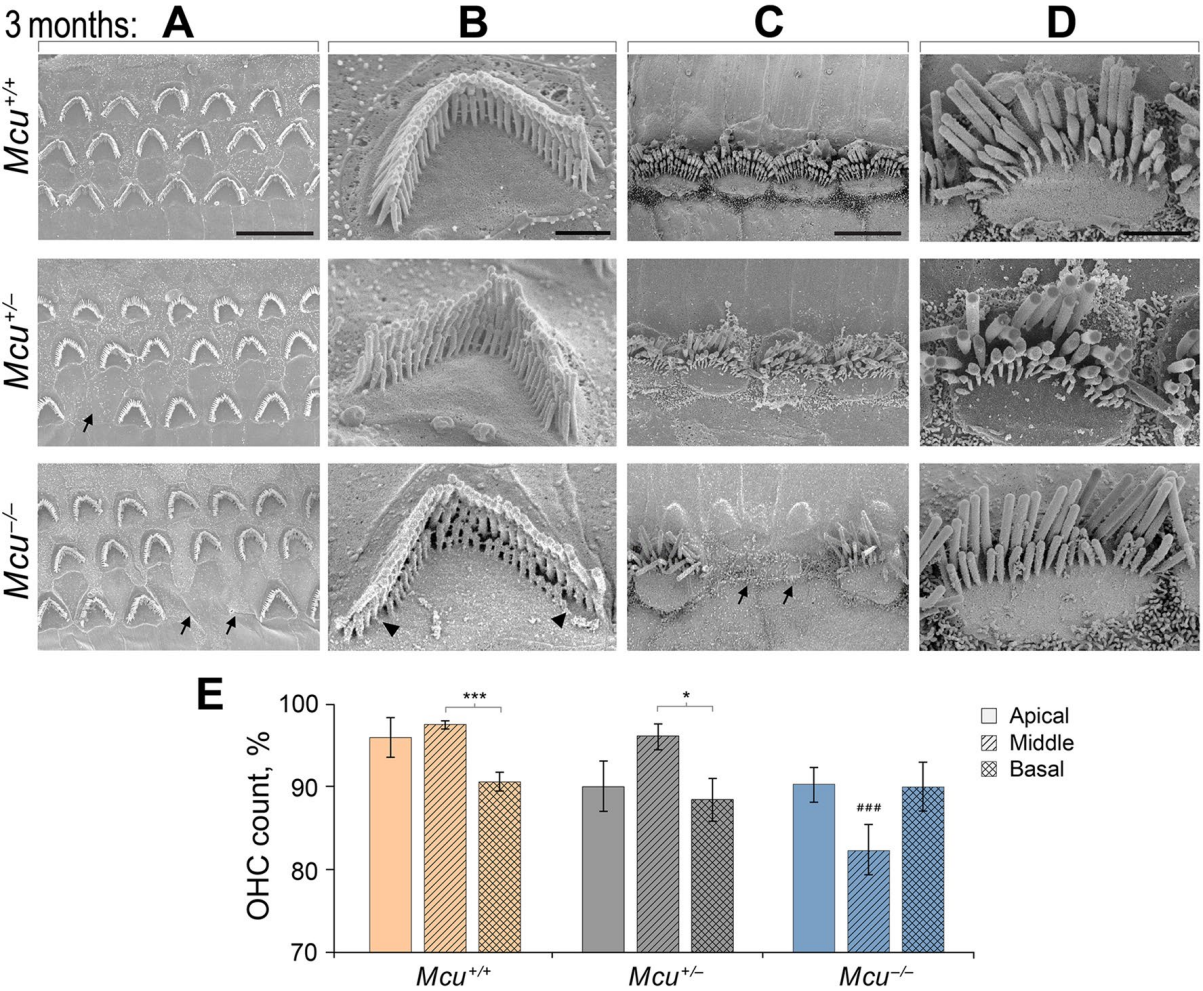
Loss of hair cells in adult *Mcu*^*−/−*^ mouse cochlea. (**A**–**D**) Representative scanning electron micrographs of cochlear hair cells acquired from *Mcu*^+*/*+^ (top row), *Mcu*^+*/−*^ (middle row), and *Mcu*^*−/−*^ FVB/NJ mice (bottom panel) at P88–P92 time point. (**A**) The typical three-row arrangement of OHCs, (**B**) single OHC stereocilia at high magnification, (**C**) the single row of IHCs, and (**D**) single IHC stereocilia at high magnification are shown. Note the absence of the third (shortest) row of stereocilia in *Mcu*^*−/−*^ mice. The arrows in (**A**) and (**C**) indicate missing sensory hair cells and the arrowheads in B indicate the degenerating shortest row of stereocilia in OHCs. (**E**) The SEM images were categorized based on their cochlear region (apical, middle, and basal turn), and the percentage of missing OHCs were plotted (mean ± SEM). ^###^*p* < 0.001 in comparison to the middle region of *Mcu*^+*/*+^ and *Mcu*^+*/−*^ mice, *p < 0.05, ***p < 0.001, Kruskal–Wallis with Dunn’s multiple comparison tests. The number of mice is 12 (*Mcu*^+*/*+^), 13 (*Mcu*^+*/−*^), and 18 (*Mcu*^*−/−*^). Scale bars are (in µm): 10 (**A**); 1 (**B**); 5 (**C**); 2 (**D**).

The elevated ABR thresholds observed at one month of age in *Mcu*^*−/−*^ mice were not paralleled by any apparent morphological and structural changes in HCs. Hence, we inspected the structural integrity of HCs at 3 months of age (Fig. 7). A fraction of the outer hair cells (OHCs) were missing and the stereociliary bundles of the OHCs that were present showed signs of degeneration in the *Mcu*^*−/−*^ mice. Specifically, the third row of hair bundles was affected by the degenerating stereocilia at the edges (Fig. 7A,B lower panel). Occasionally, a few inner hair cells (IHCs) were lost in the *Mcu*^*−/−*^ mice. IHC hair bundles of *Mcu*^*−/−*^ mice lacked the third, the shortest, row of stereocilia, in striking contrast to hair bundles of *Mcu*^+*/*+^ and *Mcu*^+*/−*^ mice (Fig. 7C,D lower panel). In the *Mcu*^+*/−*^ mice, the number of OHCs that were missing was comparably less than in the *Mcu*^*−/−*^ mice but higher than in the *Mcu*^+*/*+^ littermates (Fig. 7A, middle panel). The stereociliary bundles of both the OHCs and the IHCs appeared normal in the *Mcu*^+*/−*^ mice, and no IHCs were lost. Moreover, we enumerated the number of OHCs present in the apical, middle, and basal regions of *Mcu*^*−/−*^ cochlea compared to *Mcu*^+*/−*^ and *Mcu*^+*/*+^ littermates. This analysis revealed that the mid-cochlear region was the most susceptible in the *Mcu*^*−/−*^ mice with only 82.3% of the OHCs present in contrast to the > 96% of OHCs present intact in *Mcu*^+*/−*^ and *Mcu*^+*/*+^ littermates (Fig. 7E). Another observation was the loss of ~ 10% of OHCs in the apical region of both *Mcu*^*−/−*^ and *Mcu*^+*/−*^ mice in comparison to the corresponding value of 4% in *Mcu*^+*/*+^ littermates. However, this difference was not statistically significant. In addition, around 10% of OHCs in the basal region of the cochlea of all three genotypes were observed to be lost.

In conclusion, our data suggest that MCU and rapid uptake of Ca^2+^ is important for hearing preservation and long-term maintenance of auditory hair cells in congenic FVB/NJ mice.

## Discussion

The uniporter protein MCU traversing the inner mitochondrial membrane is a highly selective Ca^2+^ channel necessary for rapid mitochondrial Ca^2+^ uptake in intact cells^32–34^. Initially, MCU alone was reported to be sufficient for mitochondrial Ca^2+^ uptake^34^. However, further research has identified several other proteins that either act in concert with MCU or fine-tune its activity, thus forming a uniporter complex or uniplex. With pentameric MCU forming the main Ca^2+^ permeant pore that is incumbent for rapid Ca^2+^ uptake, the uniplex includes other protein components such as MCUb, EMRE, MICU1, MICU2, MICU3, MCUR1, and SLC25A23^35,36^. The gene encoding for MCU is well conserved in eukaryotes except for yeasts^34^; in humans, it is expressed in all major tissues and organs^37^. Although Ca^2+^ ions are known to regulate numerous and diverse aspects of cochlear physiology^38^, whether the gene coding for MCU is essential for hearing in mammals is yet to be demonstrated. In this study, we show that *Mcu*^*−/−*^ adult mice in FVB/NJ background progressively lose their hearing and display accelerated hearing loss leading to profound deafness by 6 months, across all frequencies tested. The *Mcu*^+*/−*^ mice displayed a less severe but gradual elevation in ABR thresholds in the mid- to high-frequency ranges. The *Mcu*^+*/−*^ auditory phenotype, which surfaces at ~ 3 months of age, reveals that both copies of *Mcu* are necessary for mice to preserve hearing in the long-term. Our results show that hearing impairment is not due to prenatal defects in cochlear development or early postnatal maturation of the cochlea. Specifically, loss of *Mcu* had no obvious effect on mechanotransduction function, Ca^2+^ homeostasis, or mitochondrial superoxide levels in the hair cells (HCs). A structural correlate to hearing loss in *Mcu*^*−/−*^ mice became apparent in adult cochlea around 3 months of age, when a significant fraction of HCs in the mid-cochlear turn showed signs of degeneration. Considering the *Mcu* knockout allele originated in the C57BL/6 background, and because of its linkage to the *Cdh23*^*ahl*^ loci, the FVB/NJ *Mcu*^*−/−*^ mice are also homozygous for the age-related hearing loss linked *Cdh23*^*c.753A*^ allele. Thus, MCU deficiency, possibly along with the *Cdh23*^*c.753A*^ allele, resulted in hearing loss and degeneration of HCs in the FVB/NJ mice.

We set out by demonstrating the progressive hearing impairment intrinsic to the mixed B6-CD1 background of *Mcu* knockout mice reported originally^17^. Our observation is concordant with the results reported by a study, where both *Mcu*^*−/−*^ and *Mcu*^+*/*+^ mice of the same mixed B6-CD1 background had elevated ABR thresholds at high frequency^18^. However, the authors did not assess ABR thresholds in mice later than 7 weeks of age. In the present study, we recorded the ABR thresholds longitudinally in mice starting as early as 3 weeks to 12 months of age, in B6-CD1 as well as FVB/NJ background. C57BL/6J and CD1 strains are genetically predisposed to progressive hearing loss^19,20^, and are known to harbor the *Cdh23*^*ahl*^ allele in their genome^23,39^. The B6-CD1 background, despite being isogenic for *Cdh23*^*ahl*^, may present with genetic variability and/or heterozygosity at innumerable loci; based on our results, this mixed background turned out to be unsuitable to study the hearing phenotype especially relating to MCU. Another logical assumption is the presence of genomic variants in the mixed background that may mask or compensate for the loss of *Mcu*. Supporting this notion, *Mcu* loss was shown to be protective to a certain degree against acoustic stress in the mixed B6-CD1 background, while in the CBA/J mice, siRNA or Ru360 mediated inhibition of MCU was protective against noise-induced OHC loss and permanent hearing impairment^18^. Our strategy to transfer the *Mcu*^*−/−*^ to an inbred strain of mice, like FVB/NJ, minimized genetic variation and aided in resolving the hearing impairment associated with *Mcu* loss. In contrast to the FVB/NJ background, the *Mcu*^*−/−*^ (homozygous) genotype in the C57BL/6J^40^ or CBA/CaJ resulted in embryonic lethality. Put together, the expression and characteristics of the phenotype associated with the *Mcu* mutation is strongly influenced by the genetic background. Studying the consequence of *Mcu* loss in different mouse strains is important to understand the spectrum of genotype–phenotype correlation or variation, and how that might relate to human subjects^41^.

The fact that the *Cdh23*^*ahl*^ allele is linked to the *Mcu* knockout allele may partly explain the accelerated hearing loss observed in the FVB/NJ double homozygous mice. However, we argue that this hearing impairment is a direct result of loss of *Mcu* based on the following observations: (i) The elevation in ABR threshold in *Mcu*^*−/−*^ mice is noticed as early as 3 weeks, a timeline when the effect of *Cdh23*^*ahl*^ allele is insignificant on hearing in several inbred mouse strains^22,42–44^. Additionally, the rapid ABR threshold elevation at 8 kHz and 16 kHz in FVB/NJ *Mcu*^*−/−*^ mice at 1 month does not correspond to *Cdh23*^*ahl*^—induced phenotype. (ii) Moreover, *Cdh23*^*ahl*^ homozygosity is shown to be necessary in the C57BL/6 mice, but not by itself sufficient to account for the hearing loss^44^; also the C57BL/6J-derived *Cdh23*^*ahl*^ allele had little effect on hearing loss in the CBA/CaJ background^44^. (iii) The threshold elevation noted at 3 months in double heterozygous mice (*Mcu*^+*/−*^, *Cdh23*^+*/ahl*^) evidently indicates the importance of *Mcu* in hearing, given the homozygosity requirement^45^ of *Cdh23*^*ahl*^. (iv) The postulated synergistic effect of *Mcu*^*−/−*^ and *Cdh23*^*ahl*^ on hearing loss in the FVB/NJ strain is not apparent in the mixed B6-CD1 background even at high frequency. The above points suggest a greater cumulative influence of the genetic background on the loss of *Mcu* rather than *Cdh23*^*ahl*^ itself. This reiterates that AHL adheres to a quantitative trait model in which a recessive or hypomorphic allele act in concert with a few other recessive/hypomorphic alleles to control the progression and severity of hearing impairment and the age of onset^46^. For instance, *Cdh23*^*ahl*^ has been shown to interact epistatically with *Atp2b2*^*dfw/*+^genotype to accelerate hearing loss, and provides an additive effect by modulating the progression of hearing loss together with *Mass1*^*frings/frings*^ or *Sod1*^*tm1Leb/tm1Leb*^ mutations^47,48^. The effect of *Cdh23*^*ahl*^ on *Mcu* gene mutations has not been studied until now opening up a possibility for further exploration. The dispositional attribution of several genes co-transferred with the congenic *Mcu*-*Cdh23* region (Fig. 3C) adds more complexity to evaluating gene-specific phenotypic and/or modifier effects.

The current study indicates that *Mcu* in congenic FVB/NJ mice is largely dispensable during the development and maturation of HCs, or cochlea in general. The number of HCs in *Mcu*^*−/−*^ mice are comparable to values in the *Mcu*^+*/*+^ cochlea. Additionally, neither the basal levels of Ca^2+^ nor mitochondrial superoxide is different in the HCs of *Mcu*^*−/−*^ mice. The main sources of Ca^2+^ influx in HCs are the Ca^2+^ permeable mechanotransduction channels located at the tips of the shorter stereocilia of the cell^49–54^, voltage-sensitive Ca^2+^ channels at the basolateral surface of IHCs, as well as additional Ca^2+^ release from internal stores^55–59^. This excess Ca^2+^ in the HCs is attenuated by several molecules such as the endogenous Ca^2+^ buffering proteins, Ca^2+^ ATPase (PMCA) pumps that extrude Ca^2+^, and organelles including mitochondria and endoplasmic reticulum that serve as Ca^2+^ reservoirs^60^. In *Mcu*^*−/−*^ cochlea, we analyzed the transcript levels of *Pmca1*, which is expressed in the basolateral membrane of IHCs, *Pmca2* predominantly expressed in the hair bundles of OHCs, and *Ocm* which codes for oncomodulin, a dominant Ca^2+^ buffer of the EF-hand superfamily in OHCs^61–63^. The transcript levels of these molecules are unchanged despite the significant loss in expression of *Mcu*. On the other hand, mitochondrial matrix Ca^2+^ is not completely abolished in the *Mcu*^*−/−*^ mice asserting the existence of additional mitochondrial Ca^2+^ channels as pointed out by several studies^17,64^. Debate continues on whether these additional channels allow rapid Ca^2+^ uptake or a gradual accumulation to sustain mitochondrial Ca^2+^ homeostasis in the absence of MCU^65^. The ability of model organisms, specifically selective strains of mice, to survive without any overt phenotype in the absence of MCU supports the idea that MCU is either dispensable or is functionally compensated^17,66–71^. Nonetheless, the crucial role of MCU in several contexts, including acute energy demand, strenuous exercise, pathological conditions, tissue bioenergetics, and longevity cannot be disregarded^11,17,68–70^. HCs and other cell types in the cochlea likely face acute energy demand after the onset of hearing, due to chronic stimulation from the surroundings. Our results confirm that cochlear development and onset of acoustic function are not significantly affected in the absence of *Mcu*. The timeline of the hearing loss phenotype observed in *Mcu*^*−/−*^ mice in this study recapitulates the crucial role of MCU in acute energy demand. To the best of our knowledge, this is the first report to show the hearing loss phenotype in *Mcu*^*−/−*^ mice.

Although unlikely, the degeneration of HCs and their stereociliary bundles in adult FVB/NJ *Mcu*^*−/−*^ mice may be the synergistic result of MCU deficiency and *Cdh23*^*ahl*^. The role of MCU in this aspect could be explained partially by the concentration of mitochondria along the lateral wall and in the belt beneath the cuticular plate of HCs^72,73^, where MCU potentially contributes to the clearance of Ca^2+^ entering from the stereocilia during acoustic stimulation^49^. The integrity of stereocilia in HCs is dependent on chronic Ca^2+^ influx via MET channels^74^, and without MCU to handle excess Ca^2+^, the stereocilia may degenerate. In addition, the presence of *Cdh23*^*ahl*^ allele disrupts the normal exon splicing of *Cdh23* and causes in-frame exon-skipping^46^. Because Cadherin 23 is a component of the tip links of hair cell stereocilia along with Protocadherin 15^75,76^, the presence of *Cdh23*^*ahl*^ variant may affect the preservation of stereocilia function contributing to HC degeneration^42,44^. However, the lack of correlation in the timeline corresponding to the onset of hearing loss and the degeneration of HCs in the FVB/NJ *Mcu*^*−/−*^ mice indicates that the latter is a secondary manifestation of the hearing deficit. Genetic mouse models carrying mutated *Otof*, *Ocm,* or *Vglut3* genes^77–79^ are good examples to show impairment of hearing function ahead of visible hair cell or stereocilia damage. Alternatively, when compared against ABR, a quantitative technique, SEM imaging may fail to capture small structural changes in hair cells that commensurate with a decline in hearing. Degeneration of stereociliary bundles of OHCs and, less frequently, IHCs in older mice, initiating at the base of the cochlea and gradually progressing to the apex, is a distinctive characteristic of the *Cdh23*^*ahl*^ allele^42,48,80^. In contrast, the HC and stereocilia degeneration observed in FVB/NJ *Mcu*^*−/−*^ mice at 12 weeks of age, predominantly along the mid-frequency region of the cochlea, might be the outcome of *Mcu* loss. A burgeoning question is why do only a middle turn fraction of HCs and their stereocilia degenerate in *Mcu*^*−/−*^ mice? While further research is warranted, the scientific community is starting to appreciate the tissue-specific or cell-type-specific functions of MCU^81^. In summary, the present study provides convincing evidence that *Mcu* is essential for the preservation of hearing and stereocilia maintenance in congenic FVB/NJ mice. Understanding the precise mechanism associated with the loss of *Mcu* and the primary site of dysfunction that lead to the observed phenotype is important. Since the FVB/NJ *Mcu* mutant mice used in this study are constitutive knockouts, it is difficult to ascertain the initial site of the genesis of hearing loss in this model. It is also possible that the observed phenotype in *Mcu* mutant mice is a synergistic effect of mitochondria-rich cell types in the cochlea (hair cells, stria vascularis, and spiral ganglion neurons) and the *Cdh23*^*ahl*^ allele. Future studies involving cell-type-specific conditional knockout mice will be necessary to dissect the mechanism of hearing loss induced by the lack of MCU.

## Methods

### Animals

The *Mcu*^+*/−*^ heterozygous mice on a mixed B6-CD1 background were purchased from the Texas A&M Institute of Genomic Medicine^17^. The FVB/NJ mice and CBA/CaJ mice were purchased from The Jackson Laboratory (Stock No. 001800 and 000654, respectively). After 6 generations of backcrossing through both the female and the male FVB/NJ line, two *Mcu*^+*/−*^ progeny were intercrossed and animals heterozygous for mutant *Mcu* were selected to generate the experimental mice. All mice had free access to regular feed and water and were maintained under the standard light–dark conditions (12:12 h) at the Animal Resource Centre of Case Western Reserve University (CWRU). An approximately equal proportion of females and males were used in all experiments. All mice experiments and research protocols were approved by the Institutional Animal Care and Use Committee (IACUC) of CWRU. The study was carried out in compliance with the ARRIVE guidelines and all methods were performed in accordance to the IACUC regulations which meets or rather exceeds the requirements of the following agencies: Department of Agriculture Animal Welfare Act, Public Health Service Policy on Humane Care and Use of Laboratory Animals, and Association for Assessment and Accreditation of Laboratory Animal Care International.

### DNA isolation and genotyping

Ear punched tissues of weaned mice were collected in sterile Eppendorf tubes and were processed for DNA isolation. Briefly, the tissues were lysed in SNET Lysis buffer, hair and other debris were removed by centrifugation, and the remaining supernatant was mixed with an equal volume of Isopropanol. DNA was pelleted by centrifugation, washed once with 70% Ethanol, and air-dried at RT for 15 min. The DNA was dissolved in 40 µL 0.5× TE buffer at 50 °C for 1 h and then 2 µL of each of the sample was used for PCR genotyping with DreamTaq Green PCR Master Mix (Thermo Fischer Scientific), and the following primers:

*Mcu* WT Fwd: 5′-GGAGTTAAGTCATGAGCTGC-3′.

*Mcu* WT Rev: 5′-CTGGCTTAGTTGGCAGAGTT-3′.

*Mcu* KO Rev: 5′-GACTTGTGGTCTCGCTGTTC-3′.

The primers were synthesized by Integrated DNA Technologies and were used at a final concentration of 200 nmol/PCR reaction. The total reaction volume of the PCR was 20 µL, and the conditions were initial denaturation at 94 °C for 5 min., followed by 35 cycles of 94 °C for 15 s, 48 °C for 15 s, and 72 °C for 1 min, followed by a final extension at 72 °C for 10 min and 4 °C hold. The wild type allele of *Mcu* produced amplicons of 311 bp while the KO allele amplicons were 176 bp in length. The PCR products were resolved in a 1.5% Agarose Gel run in 1X TAE at 100 V, 400 mA for 35 min. Bullseye DNA SafeStain (MidSci) was used to visualize the DNA.

We developed a PCR–RFLP (Restriction Fragment Length Polymorphism) method for genotyping the *Cdh23*^*c.753A/G*^ polymorphism. A segment of exon 7 spanning the single nucleotide variant of interest of *Cdh23* was amplified using the following primers:

*Cdh23*.E7-Fwd 5′-AAGCTGTGTCATTATGTGTGGTAC-3′.

*Cdh23*.E7-Rev 5′-TACTGTAGCACCATCAGGCTC-3′.

PCR was set up to a total volume of 20 µL, with 100 nmol final concentration of each of the primers, 100 ng of genomic DNA and 1× DreamTaq Green PCR Master Mix. The PCR conditions were 95 °C for 2 min, followed by 40 cycles of 95 °C for 30 s, 60 °C for 30 s, and 72 °C for 30 s, a final extension of 72 °C for 2 min, and 10 °C hold. 10 µL of the PCR products were run in 1.5% agarose gel to verify amplification, and the remaining 10 µL was subjected to restriction digestion using 6 units of *BsrI* restriction enzyme (New England Biolabs) in 1× NEB 3.1 buffer totaling to a final volume of 25 µL. The digestion was performed at 65 °C for 1 h, followed by inactivation at 80 °C for 20 min. and 10 °C hold. Following digestion, the products were resolved in a 1.5% agarose gel with undigested controls and 100 bp DNA ladder. The 534 bp amplicons with *Cdh23*^*c.753G*^ allele remain undigested with *BsrI*, while the *Cdh23*^*c.753A*^ allele produces two fragments of 350 bp and 184 bp upon *BsrI* digestion. Validation was carried out by sequencing the PCR amplicons to verify restriction digestion.

### Auditory brainstem response

The mice were weighed, and anesthetized with an Intra-peritoneal (IP) injection of a cocktail containing ketamine (20 mg/mL) and xylazine (1.75 mg/mL) at a dose of 0.1 mL/20 g. The depth of anaesthesia was tested by the toe-pinch response, eyes lubricated by application of PURALUBE^®^ VET Ointment, and the mice were placed in a sound-isolated chamber. The body temperature was maintained at 37 °C through a temperature-controlled heating pad. Subdermal electrodes were inserted at the vertex of the skull (positive), the mastoid region under the left ear (ground), and the mastoid region under the right ear (negative). Pure tone bursts were delivered through earphones using a computer-aided evoked potential system (Intelligent Hearing Systems, the Smart-EP software 3.30, Miami, FL, USA). Thresholds were determined for the 8 kHz, 16 kHz, and 32 kHz frequencies by escalating or deescalating the intensity in 10-dB steps starting at 70 dB, and then in 5-dB steps near-threshold until no organized responses were detected. For each stimulus, 768 sweeps were averaged for a response. Thresholds were defined as the lowest stimulus level where an organized response is observed. The recordings were performed blindly without knowledge of the genotype, and later the recorded values were matched with the genotype and grouped for statistical analyses.

### RNA extraction and quantitative PCR

The mice cochleae were quickly dissected and isolated in ice-cold PBS, transferred to RNA*later*™ solution (Thermo Fischer Scientific), and stored at 4 °C for a day. The next day, cochleae were transferred to ice-cold PBS to give a quick wash, and TRIzol (Invitrogen, Eugene, OR, USA) was used for total RNA extraction following the manufacturer's recommendation and instructions. The SuperScript IV First-Strand Synthesis System (Invitrogen) was utilized to synthesize cDNA from 2 µg of total RNA and used subsequently at a final concentration of 10 ng/reaction in qPCR. Quantitative PCR was performed using the PowerUp SYBR Green Master Mix (Thermo Fischer Scientific, A25742) on an ABI StepOnePlus™ Real-Time PCR System (Thermo Fischer Scientific). Details of the primers are provided in Table 1. The cycle reaction was performed as follows: 50 °C for 2 min, 95 °C for 2 min, 40 cycles of 95 °C for 15 s and 60 °C for 1 min, and finally a dissociation curve of 95 °C for 15 s and 60 °C for 15 s. The gene expression was calculated relative to the housekeeping genes *Hprt* and *PolR2f* and then analyzed using the 2^−ΔΔCT^ method^82^.

**Table 1:** Details of qPCR-primers.

Gene	Amplicon length (in bp)	Primer name	Primer sequence (5′ → 3′)
*Mcu*	187	*Mcu*_Fw	GTTGTGCCCTCTGATGACG
		*Mcu*_Rev	CTGGTGAGTAGATGGCGAC
*Pmca1*	193	*Pmca1*_Fw	ACCTGTAGCTGACATTACTGTTG
		*Pmca1*_Rev	ACCTTCCATCACATGAGTACCT
*Pmca2*	103	*Pmca2*_Fw	CTGTGCAGATAGGCAAGGC
		*Pmca2*_Rev	CATGGCTTCTTGTTGACCACG
*Ocm*	161	*Ocm*_Fw	AGCATCACGGACATTCTGAGC
		*Ocm*_Rev	TGGTCGTTGTCTATGAACTGGA
*Hprt*	123	*Hprt*_Fw	CTCATGGACTGATTATGGACAGGAC
		*Hprt*_Rev	GCAGGTCAGCAAAGAACTTATAGCC
*PolR2f*	101	*PolR2f*_Fw	CGACGACTTTGATGACGTTG
		*PolR2f*_Rev	GCTCACCAGATGGGAGAATC

*Mcu* mitochondrial calcium uniporter, *Pmca* plasma membrane calcium ATPase, *Ocm* oncomodulin, *Hprt* hypoxanthine phosphoribosyltransferase, *PolR2f* polymerase (RNA) II (DNA directed) polypeptide F, *Fw* forward primer, *Rev* reverse primer.

### The organ of Corti explants, imaging and analysis

Organ of Corti explants were obtained as described previously^24–26^. Organs were dissected at postnatal days 3–4 (P3–P4). Briefly, the sensory epithelium was separated first from the lateral wall and then from the modiolus. The most basal “hook” region was cut out and the remaining organ was placed into a glass-bottom Petri dish (FluoroDish, WPI) and cultured in DMEM medium supplemented with 7% fetal bovine serum (Invitrogen, Carlsbad, CA) at 37 °C and 10% CO_2_. The organ of Corti explants were kept in vitro from one to five days before the experiments. The equivalent age (age of dissection plus days in vitro) of the specimens reported in this study was P5–P9. To prevent contamination, 10 µg/mL of ampicillin (Calbiochem, La Jolla, CA) was added to the medium. FM1-43 (Invitrogen) was used to evaluate mechanotransduction and to count functional hair cells. FM1-43 was dissolved in DMSO to obtain a stock solution with a concentration of 1 mM. Immediately before the experiment, the stock solution was diluted to 5 μM in Ca^2+^-free HBSS. Then, all specimens were briefly incubated in FM1-43 for 30 s at room temperature and then carefully rinsed in standard HBSS. MitoSOX Red superoxide indicator (Invitrogen), as well as calcium indicators Fluo-2 LeakRes AM (TEFlabs) and Rhod-2 AM (Biotium), were used according to manufacturer provided protocol. The raw images were gathered using an upright Olympus BX51WI microscope equipped with a 100 × 1 NA objective and a Grasshopper3 CMOS camera (FLIR), using manufacturer provided software. Images were subjected to fluorescence measurements using ImageJ (NIH). A region of interest was used to obtain measurements from the hair cells (I_cell_) and an area without cells (I_background)_ in the same image. Fluorescence intensity (I_load_) for the hair cells were normalized (I_load_ = I_cell_ − I_background_).

### Scanning electron microscopy

Samples were prepared as previously described^25^. Temporal bones of mice at the age of 4 weeks and 12 weeks were removed after euthanasia by CO_2_ inhalation and fixed with 2.5% glutaraldehyde in 0.1 M sodium cacodylate buffer, pH 7.4, (Electron Microscopy Sciences, Hatfield, PA), supplemented with 2 mM CaCl_2_. After removing the stapes, and opening the round and oval window, the cochleae were immersed in the fixative for 2 h and then stored at 4 °C in 1/10th fixative diluted with buffer until collected for further preparation. The cochleae were removed, then washed with PBS, and placed in 5% EDTA, pH 7.4 at 4 °C for three days to decalcify. Following decalcification, samples were dissected and organs of Corti were removed and divided into apical, middle, and basal parts. The specimens were then dehydrated in a gradient ethanol series, critical-point dried using CO_2_, mounted on an SEM stub, and sputter-coated with 10 nm palladium. Cochlear epithelia were viewed and imaged with a high-resolution scanning electron microscope (FEI Helios NanoLab 650, Germany) housed at Swagelok Center for Surface Analysis of Materials (SCSAM), CWRU.

### Statistical analysis

All statistical analyses were performed using Microsoft Excel and GraphPad Prism 7. Data are reported as mean ± SEM unless noted otherwise. Two way ANOVA analyses with post hoc pairwise comparisons using Bonferroni adjustments or Kruskal–Wallis test with Dunn’s multiple comparisons were used depending on data distribution. Comparisons between two groups were tested by Student’s *t*-test. P values < 0.05 were considered significant.
